# A quantitative comparison of essential cardiovascular medicines from countries in the Southern African Development Community to the WHO model essential medicines list

**DOI:** 10.1186/s40545-022-00494-0

**Published:** 2022-12-08

**Authors:** Ian Naicker, Fatima Suleman, Velisha Ann Perumal-Pillay

**Affiliations:** 1 Discipline of Pharmaceutical Sciences, University of KwaZulu-Natal, Private Bag X54001, Durban, 4000 South Africa; 2 College of Health Sciences, University of KwaZulu-Natal, Durban, South Africa

**Keywords:** Essential medicines list, Cardiovascular diseases, South Africa, Southern African Development Community (SADC), World Health Organization

## Abstract

**Background:**

Globally, cardiovascular disease (CVD) is a leading cause of death and disproportionately affects low- and middle-income countries (LMICs). The WHO Model List of Essential Medicines (WHO EML) is a tool for improving accessibility and availability of medicines. This study compared the 2021 WHO EML CVDs basket of medicines with latest available national essential medicines list (NEMLs) for South Africa and 15 Southern African Development Community (SADC) countries to assess consistency in CVDs medicine listing.

**Methods:**

This descriptive, desktop review study compared SADC NEMLs. A comparator list was extracted by combining cardiovascular medicines listed in the 2021 WHO EML for adults and children. SADC country NEMLs were obtained from the WHO Essential Medicines and Health Products Information Portal. Consistency of NEMLs was calculated as a percentage coverage of CVD medicines listed in the 2021 WHO EML. SA hospital and primary health care (PHC) level NEMLs were included as separate formularies.

**Results:**

The SA hospital level NEML scored 70% consistency with the 2021 WHO EML. Tanzania (84%), Namibia (81%) and Angola (79%) scored the highest consistency. The mean consistency for SADC NEMLs was 66%. The lowest scoring country was Eswatini at 26%. The SA PHC NEML scored 35%. The least listed medicines were beta-blockers, angiotensin receptor blockers (ARBs), clopidogrel (43%) and paediatric formulations (furosemide (21%); digoxin (43%)). Individual antihypertensive medicines were most commonly listed. Botswana and Lesotho were the only countries to list a single pill combination (SPC) for the treatment of hypertension.

**Conclusions:**

This comparison indicates that South Africa and most SADC countries are aligned with 2021 WHO EML recommendations. The inclusion of age-appropriate formulations for children as well as ARBs and SPC for the treatment of hypertension may improve patient adherence and cardiovascular outcomes in these countries. More frequent updates to NEMLs should improve consistency. NEMLs were not available for two countries, and these therefore did not form part of this study. Country health expenditure in ranking the consistency of NEMLs was not accounted for. LMICs adopting the essential medicine list strategy should consider imposing minimum consistency thresholds to the WHO EML to improve accessibility and availability of CVD medicines.

*Trial registration*: Not applicable.

**Supplementary Information:**

The online version contains supplementary material available at 10.1186/s40545-022-00494-0.

## Background

Cardiovascular disease (CVD) is currently the leading cause of death in the world and disproportionately affects low and middle-income countries [1, 2]. Sub-Saharan Africa (SSA) is currently facing a threat of cardiovascular disease of epidemic proportions. There was an estimate of one million deaths in 2013 alone with the burden of CVDs predicted to double by 2030 [3]. This increase in CVDs has been attributed to economic, nutritional, demographic, and epidemiological transitions [4].

South Africa is in a state of advanced epidemiological transition with an epidemic of infectious diseases and a concurrent increase in non-communicable diseases (NCDs) [5]. CVDs are the leading cause of death in South Africa after HIV/AIDS and are responsible for every one in six deaths (17.3%). Hypertension is the leading risk factor for death from CVDs in South Africa. Blood pressure lowering medications have proven efficacy in controlling hypertension and reducing associated CVD events, but their effectiveness has been suboptimal throughout sub-Saharan Africa due to barriers like availability and cost of medicines [6].

Accessibility and availability of essential medicines are a key strategy to improving CVD prevention and control [7]. The WHO defines essential medicines as those that satisfy the priority health care needs of a population. They are selected based on disease prevalence and public health relevance, clinical efficacy and safety as well as comparative costs and cost-effectiveness. Essential medicines are intended to be available at all times in adequate amounts and in appropriate dosage forms [8]. The WHO has developed, and regularly updated, a model list of essential medicines to be used as a guide for the development of national and institutional lists primarily for low- and middle-income countries. The WHO Model List of Essential Medicines (WHO EML) is a fundamental tool for improving accessibility and availability of medicines to prevent and reduce CVDs [9]. The first WHO EML was published in 1977 and is revised every 2 years. At the time of this research, the WHO EML is in its 22nd edition and the uptake of the model list has increased over time with 131 countries having officially adopted the tool in 2020 [8].

The South African National Essential Medicines List (NEML) was first published in 1996 and there have been 12 editions since then. The NEML in South Africa is segmented into three separate lists: that of primary health care (PHC), hospital level adult and paediatric care. Essential medicines are extracted from standard treatment guidelines (STGs) by the National Department of Health (NDOH) [10].

**Fig. 1:**
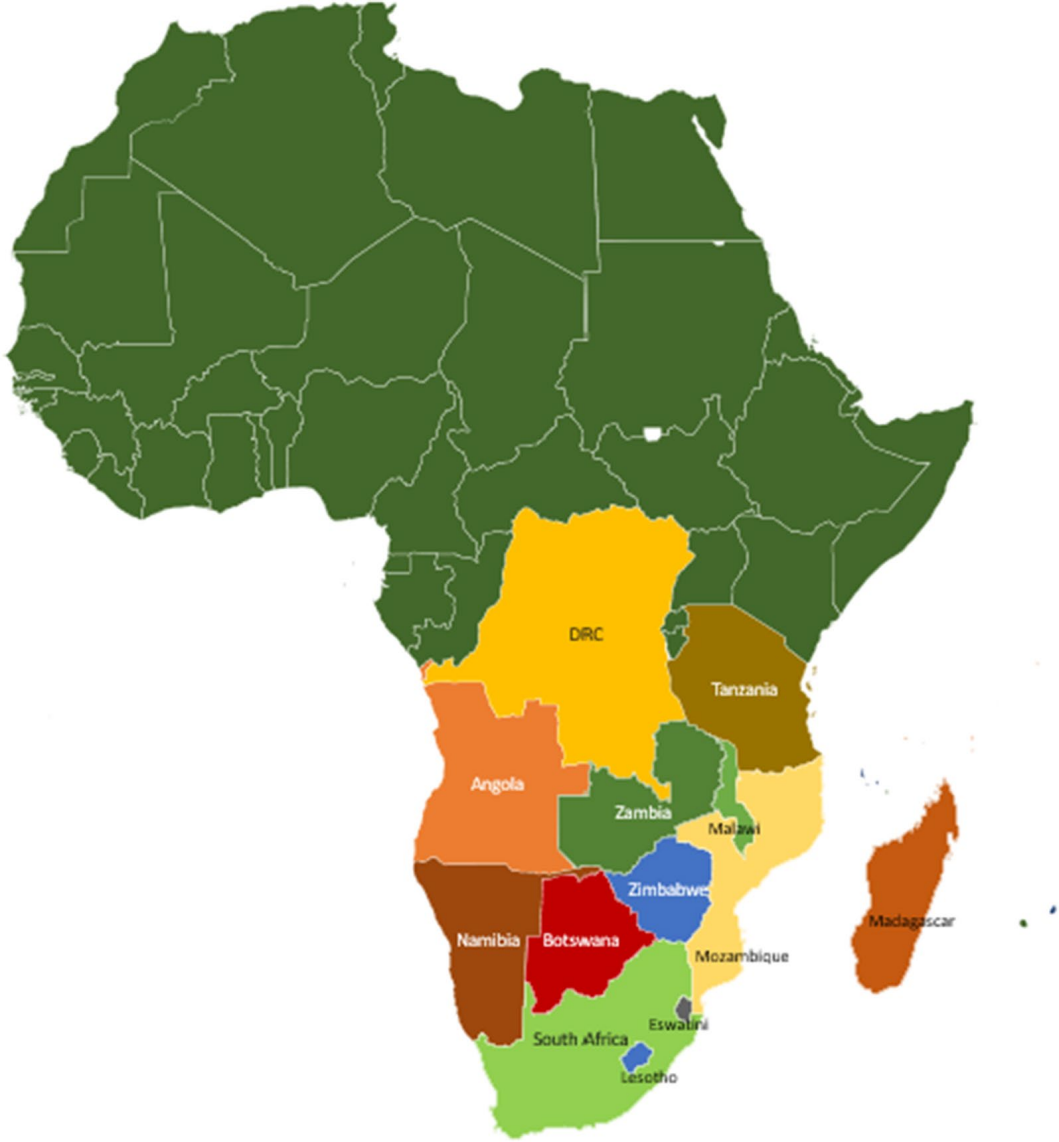
Southern African development community (SADC) countries in Africa. (The Republic of Mauritius and Federal Islamic republic of the Comoros not included due to scale.)

The objective of this study was to compare the 2021 WHO EML for the treatment and prevention of CVDs to the South African NEML and NEMLs from selected regional countries in the Southern African Development Community (SADC—Fig. 1). This is a regional economic community comprising 16 member states. These are Angola, Botswana, Comoros, Democratic Republic of Congo, Eswatini, Lesotho, Madagascar, Malawi, Mauritius, Mozambique, Namibia, Seychelles, South Africa, Tanzania, Zambia and Zimbabwe.

There have been few studies that have compared NEMLs with the WHO EML with a specific focus on cardiovascular medicines. In 2019 Persaud and colleagues compared NEMLs of 137 countries to the 2017 WHO EML but did not focus specifically on cardiovascular diseases [11]. In 2021, Steiner et al. used this database to determine the relationship between the listing of essential medicines and cardiovascular outcomes [12]. Barzagani et al. explored the selection of medicines used to treat cardiovascular diseases in 35 low- and middle-income countries which included selected African countries [13]. Mehrtash and colleagues completed a cardiovascular focused analysis of the NEML of countries in the Eastern Mediterranean region and compared them to the 2015 WHO EML [14]. In 2020, Dzudie et al. looked at the availability, cost and affordability of essential CVD medicines in Cameroon [15]. However, to date there have been no studies that have focused on comparing the list of essential CVD medicines from a subset of sub-Saharan African countries to the 2021 WHO EML. We also included the 2021 WHO EML for children in our study. A comparison of combined adult and paediatric medicines was not previously undertaken.

## Methods

The objective of the study was to quantitatively compare cardiovascular medicines on the South African NEML for adults and children with the 2021 WHO model list of essential medicines. The study also compared essential medicines lists from neighbouring countries from the southern African region with the 2021 WHO model list of essential medicines in respect of cardiovascular medicines. For this regional comparison countries that belong to the Southern African Development Community were selected. The study employed a descriptive quantitative design using observational data from a desktop review.

### Creating a comparator list

A list was extracted combining medicines listed under ‘cardiovascular medicines’ in Sect.  12 of the 2021 WHO EML for adults and children. This served as our comparator list (Table 1). The combined 2021 WHO EML for the treatment of CVDs consisted of 43 medicines. The paediatric component contributes four medicines in eight dosage forms to this list. Cardiovascular medicines were classified into six categories viz. antianginal medicines; antiarrhythmic medicines; antihypertensive medicines; heart failure medicines; antithrombotic medicines and lipid lowering agents.

**Table 1:** Combined comparator list of adult and child cardiovascular medicines extracted from 2021 WHO model list of essential medicines

Medication	Dosage form	Comments
**Adults**		
Antianginal medicines		
Bisoprolol	Tablet	Alt^a^: metoprolol; carvedilol
Glyceryl trinitrate	Tablet/SL^b^	
Isosorbide dinitrate	Tablet/SL	Class effect^c^
Verapamil	Tablet	Hydrochloride
Antiarrhythmic medicines		
Bisoprolol	Tablet	Alt: metoprolol; carvedilol
Digoxin	Tablet/ injection	
Epinephrine	Injection	10 ml ampoule; HCL or acid tartrate
Lidocaine	Injection	5 ml ampoule; HCL
Verapamil	Tablet/injection	HCL^d^
Amiodarone	Tab/injection	3 ml ampoule; HCL (comp)
Antihypertensive medicines		
Amlodipine	Tablet	Maleate, mesylate, besylate; class effect
Bisoprolol	Tablet	Alt: atenolol; metoprolol; carvedilol
Enalapril	Tablet	Hydrogen maleate; class effect
Hydralazine	Tablet	HCL; pregnancy-induced hypertension
Hydrochlorothiazide	Tablet	Alt: chlorothiazide; chlorthalidone; indapamide
Lisinopril + amlodipine	Tablet	Single pill combination therapy; class effect
Lisinopril + hydrochlorothiazide (HCT)	Tablet	Single pill combination therapy; class effect
Losartan	Tablet	Class effect
Methyldopa	Tablet	Pregnancy-induced hypertension
Telmisartan + amlodipine	Tablet	Single pill combination therapy; class effect
Telmisartan + HCT	Tablet	Single pill combination therapy; class effect
Sodium nitroprusside	Powder for infusion	Complementary
Heart failure medicines		
Bisoprolol	Tablet	Alt: metoprolol; carvedilol
Digoxin	Tablet	
Enalapril	Tablet	Hydrogen maleate; class effect
Furosemide	Tablet/injection	Class effect
Hydrochlorothiazide	Tablet	
Losartan	Tablet	Class effect
Spironolactone	Tablet	
Dopamine	Injection	5 ml vial; HCL; complementary listing
Antithrombotic medicines		
Anti-platelet medicines		
Acetylsalicylic acid	Tablet	
Clopidogrel	Tablet	
Thrombolytic medicines		
Alteplase	Powder for inj	Vial; complementary listing
Streptokinase	Powder for inj	Vial; complementary listing
Lipid lowering agents		
Simvastatin	Tablets	Use in high-risk patients; class effect
**Children**		
Antihypertensive medication		
Enalapril	Tablet	Hydrogen maleate, class effect
Enalapril	Suspension	Class effect
Heart failure medicines		
Digoxin	Injection	2 ml ampoule
Digoxin	Oral Liquid	
Digoxin	Tablet	
Furosemide	Injection	2 ml ampoule; class effect
Furosemide	Oral liquid	
Furosemide	Tablet	
Dopamine	Injection	5 ml vial; HCL; complementary listing

^a^Alt: recommended alternatives only

^b^SL: sublingual

^c^Class effect: recommends any medicine from the same class as that of the listed medicine

^d^HCL: hydrochloride

### Extraction of data from SADC NEMLs

**Fig. 2:**
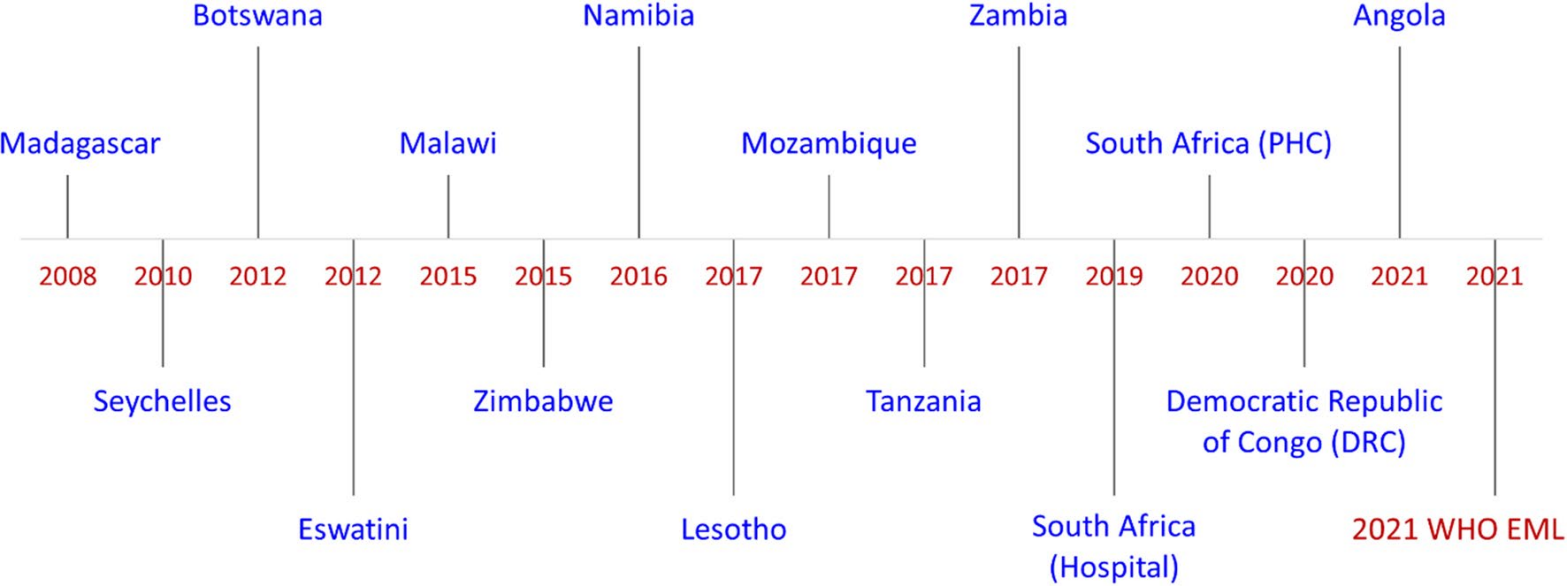
Latest available versions of NEMLs from SADC countries

The WHO Essential Medicines and Health Products Information Portal was then used to search for the national essential medicines lists of SADC countries inclusive of South Africa. In an attempt to use the latest versions of the country EMLs a further desktop search was employed to source more updated guidelines that were not included in the portal. Most of the NEMLs were in the form of STGs which could be compared with the indications of the 2021 WHO EML while others were in the form of formularies. Figure 2 shows the latest available versions of NEMLs from SADC countries. Mauritius and Comoros have not developed a NEML and could not be included in the study.

Both boxed and unboxed recommendations of the 2021 WHO EML were accounted for. Only classes of medicines that were in the 2021 WHO EML cardiovascular section were included, including those listed as complementary. Dosage form was only considered for paediatric patients. The list mirrored the indications listed in the 2021 WHO EML, so some medicines were listed more than once if they had more than one indication. Some medications were listed both in the adults and children’s section in accordance with the recommendations of the 2021 WHO EML. The availability of an essential medicine was marked with a highlighted square box and the absence of an essential medicine was marked with a cross. Additional medicines that were available on the NEMLs were also noted on the table and assigned an alphabetical superscript to assist in identification and differentiation of multiple medicines from each class (Additional file 1: Table S2).

### Calculating consistency with the 2021 WHO EML

For the purposes of this research, we calculated the consistency (CS) which represented the extent to which an NEML conforms to the 2021 WHO EML. A consistency score was calculated for each country NEML to represent the percentage coverage of the 2021 WHO EML for the treatment and prevention of CVDs. A score of 100% indicated that all the medicines in the 2021 WHO EML were represented in the country NEML.

The consistency was calculated as follows:

If there are 43 essential cardiovascular medicines in the 2021 WHO EML for adults and children, this will serve as a denominator for the consistency calculation.

Consistency (CS) = (*n*)NL / (*N*) WML × 100,

where (*n*)NL = number of medicines on the NEML and, (*N*) WML = number of medicines on the WHO Model List.

If a country had 20 medicines on this list, then the CS was calculated as follows: 20/43 × 100 = 45%. The WHO had included unrestricted and restricted square box medicines in the 2021 formulary. Unrestricted square box medicines are those that represent an entire class and are interchangeable. Restricted square box medicines are those that are interchangeable only to specified medicines from the same class [16]. The study took into account restricted and unrestricted square box medicines when calculating the CS. The study also reported on the number of medicines to prevent and treat CVDs on each list as well observed differences between the lists.

## Results

**Fig. 3:**
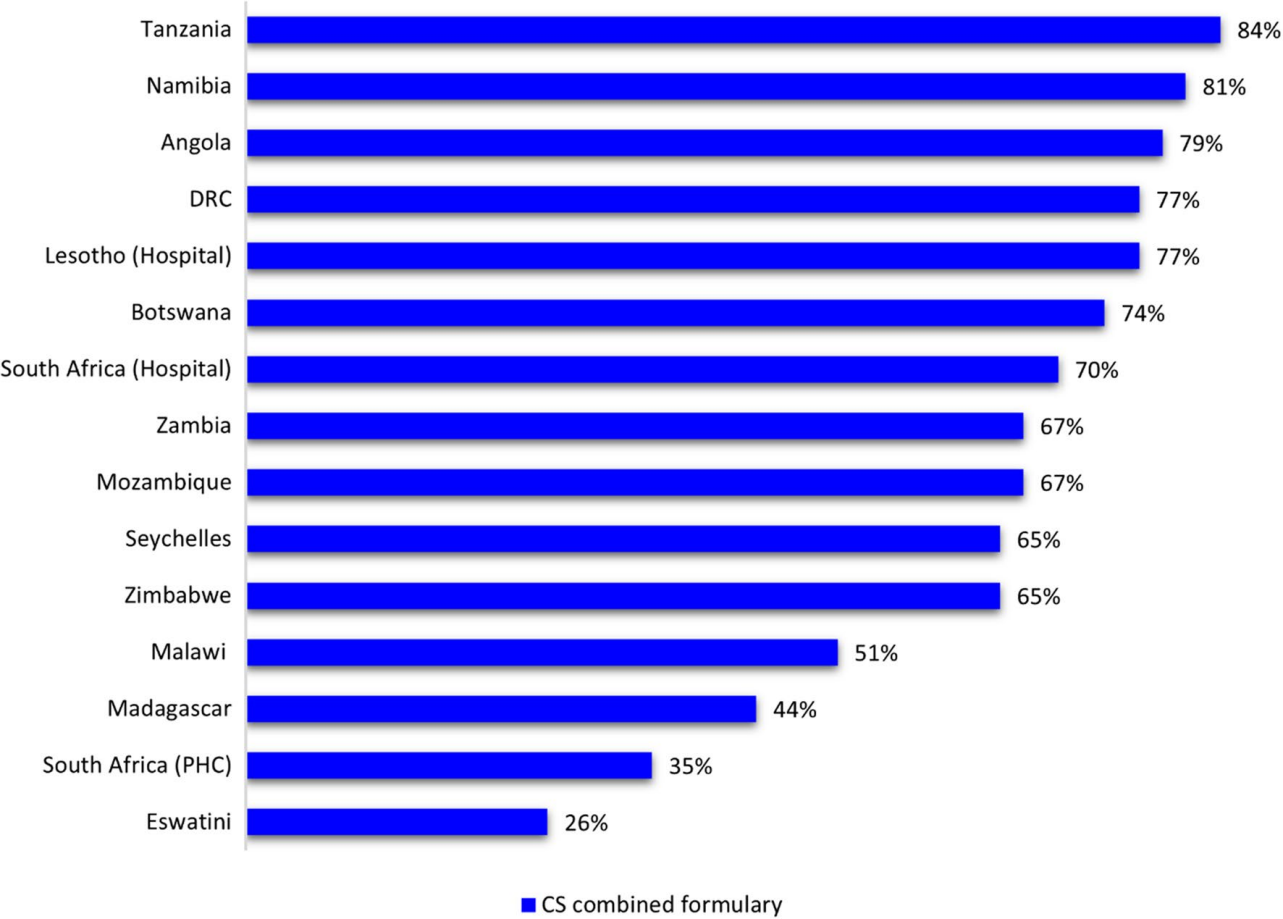
Consistency of NEMLs of the SADC countries with the 2021 WHO EML for cardiovascular diseases (SADC countries are ranked in consistency to the combined formulary)

Figure 3 illustrates the comparison of cardiovascular essential medicines lists from 16 countries identified as part of the Southern African development community with the 2021 WHO EML. National essential medicines lists from six of the fourteen countries (South Africa, Lesotho, Malawi, Eswatini, Tanzania and Zimbabwe) were in the form of standard treatment guidelines. NEMLs from the other eight countries were in the form of medicine formularies. Essential medicines lists for the island nations of Mauritius and Comoros were not available.

The overall mean consistency to the combined formulary was 66% and the median consistency was 69%. The Tanzanian NEML had the highest consistency of 84% to the combined 2021 WHO EML. The Kingdom of Eswatini had the lowest consistency from all formularies with a score of 26%. The South African NEML scored 70% consistency at hospital level and 35% at primary care level. South Africa’s NEML for hospitals ranked seventh in terms of consistency after Tanzania, Namibia and Angola.

**Fig. 4:**
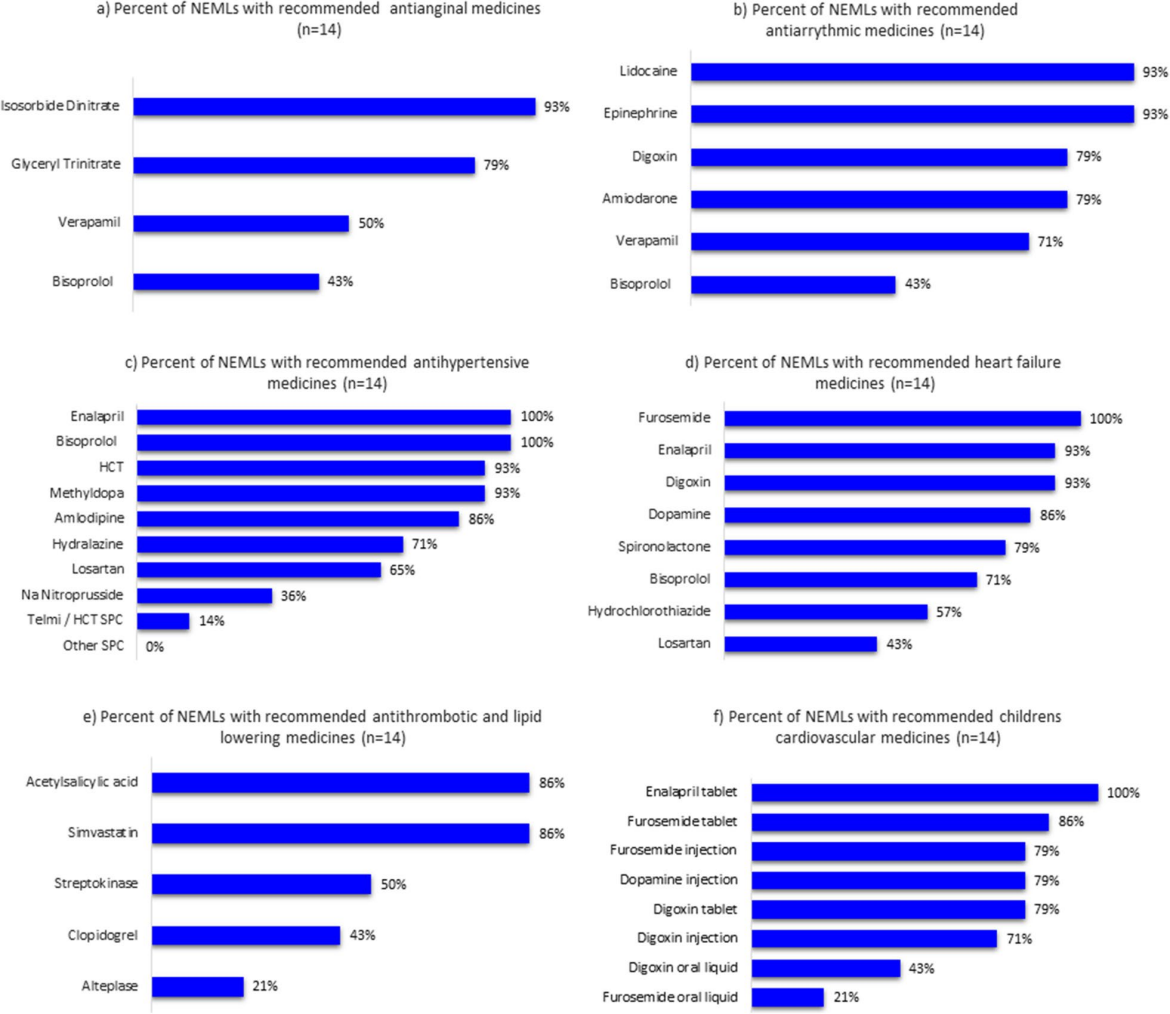
Percentage of NEMLs from SADC with 2021 WHO EML recommended medicines for cardiovascular diseases. **a**–**f** Percentage of SADC NEMLs with 2021 WHO recommended medicines for angina, hypertension, arrhythmias, heart failure, thrombosis, hyperlipidaemia and CVD medicines for children. Figures are in order of indication in the 2021 WHO EML

Bisoprolol and recommended alternatives (metoprolol and carvedilol), were the least recommended as antianginal and antiarrhythmic medicines at 43% (Fig. 4a, b). Verapamil was recommended by only 50% of the NEMLs as an antianginal medicine. Amiodarone was only listed as a complimentary medicine for the treatment of arrhythmias but was recommended by 79% percent of the NEMLs.

The medicines recommended by 2021 WHO EML for the treatment of hypertension were unrestricted square box and interchangeable with any other medicine from the same class with the exception of thiazide diuretics which were restricted to three alternatives. ACE inhibitors and beta-blockers were listed in all NEMLs for the treatment of hypertension (Fig. 4c). Angiotensin receptor blockers (ARBs) were listed in 65% of the NEMLs. The four listed single pill combinations on the 2021 WHO EML were not available in any of the NEMLs with the exception of Botswana and Lesotho who listed a single pill combination of an ARB and a thiazide diuretic on their NEMLs. The 2021 WHO EML lists both hydralazine and methyldopa for the management of pregnancy-induced hypertension. All SADC countries list either hydralazine, methyldopa or both for the treatment of pregnancy-induced hypertension. Methyldopa is only listed on the hospital level of the South African NEML.

The most commonly listed medicines for heart failure were digoxin, ACE inhibitors and loop diuretics (Fig. 4d). Although commonly listed for treatment of hypertension, just over half the guidelines listed thiazide-like diuretics for the treatment of heart failure. Only 43% percent of countries listed ARBS for the treatment of heart failure. The most common listed antithrombotic medication was aspirin while listing of clopidogrel, streptokinase and alteplase was not commonly listed. Statins were listed in 86% of the NEMLs (Fig. 4e).

Results from the paediatric basket of medicines (Fig. 4f) found that dosage forms already available for adults were most often listed, e.g., enalapril and furosemide tablets. Less than 50% of NEMLs specifically included oral liquids for digoxin (43%) and furosemide (21%) in their formularies. The South African NEML did not specify the paediatric dosage forms as recommended in the 2021 WHO EML.

Dopamine had the highest listing of complementary medicines occurring in above 80% of SADC NEMLs while amiodarone was also well listed in 79% of NEMLs. Sodium nitroprusside (36%) and alteplase (21%) were not commonly listed. The SA NEML included all complementary medicines at hospital level except for sodium nitroprusside. Streptokinase was the only listed complementary medicine on the SA PHC EML. The study also looked at the major cardiovascular drug classes listed on the SADC EMLs irrespective of which individual medicines were listed on the 2021 WHO EML. ACE inhibitors, beta-blockers, thiazide and loop diuretics appeared in 100% of SADC NEMLs. Nitrates were listed in over 90% of NEMLs while statins, MRAs and CCBs appeared in over 70% of lists. ARBs were listed in only 65% of SADC NEMLs. Alpha blockers were listed on 36% of NEMLs despite not being listed on the 2021 WHO EML.

## Discussion

South Africa and majority of SADC countries scored well in terms of consistency with the 2021 WHO EML for combined lists of CVD medicines. There have been no previous similar studies in SADC to compare with and very few studies from LMICs across the world that focused solely on cardiovascular medicines [13, 14, 17, 18]. In comparison to a similar study done in the middle east [14], South Africa and other SADC countries averaged higher in terms of consistency with the 2021 WHO model EML for combined lists of CVD medicines (66% vs 55%). While countries like Tanzania and Namibia scored very high in consistency others like Eswatini scored very low and showed similar consistency to PHC formularies. The Republic of Mauritius and the Federal Islamic Republic of the Comoros were the only two countries in SADC that did not have an official national essential medicines list. A medicines pricing and availability study in the Comoros revealed that medicine prices were more than eighty three times that of international reference prices with poor availability in the public sector [19] demonstrating the need for adopting an EML strategy in these countries.

### How does South Africa compare overall?

The South African NEML is the only formulary on the list of countries from SADC that is available in standard treatment guidelines (STGs) at a primary care level, hospital level for adults as well as for children. The advantages of STGs are, however, limited to the strength of the recommendations in the guidelines [20]. South Africa has a comprehensive STG for paediatrics which places added priority to the needs of children. This is not available in other SADC countries and many developing countries in general [11]. The South African NEML was last updated in 2017, 2019 and 2020 for the hospital level for children, hospital level for adults and primary health care, respectively. The 2019 update at hospital level occurred after the 2019 WHO EML revision. With the exception of a closed box recommendation for thiazide diuretics, the subsequent 2021 revision of the WHO EML remains unchanged from the 2019 WHO EML for cardiovascular medicines. Thus, the strength of the treatment recommendations in these STGs is contemporary with the latest revisions of the 2021 WHO model list of essential medicines for adults and children.

The consistency of the South African PHC NEML is limited to the services provided at primary health care clinics. Since CVD outcomes are highly dependent on primary health care services [20, 21], the low consistency of the South African primary health care EML could be a potential barrier in the prevention of adverse cardiovascular outcomes from CVDs.

### Antianginal and antiarrhythmic medicines

SADC country NEMLs had a high consistency with the 2021 WHO EML for medicines used to treat angina and arrhythmias. However, there was a low consistency for the recommended beta-blockers (bisoprolol, metoprolol and carvedilol) which were listed in less than half of SADC NEMLs. Instead, there was a preference for atenolol which was removed from the WHO EML for the treatment of angina and arrhythmias more than a decade ago [22]. The South African NEML also lists atenolol for the treatment of angina and arrhythmias. However, carvedilol is listed for rate control in the treatment of congestive cardiac failure only. Previous studies in LMICs also showed that atenolol was listed in preference to the WHO recommended beta-blockers in these countries [13]. The persistence of atenolol on the NEMLs for so many years could be related to pricing and availability of bisoprolol and other WHO recommended beta-blockers.

### Antihypertensive medicines

Hypertension is the foremost CVD in SSA with an estimated prevalence of around 30% [23]. In South Africa, Basu and colleagues (2019) determined that treatment with antihypertensive medicines and statins should be prioritised above glucose lowering treatments [24, 25].

SADC country NEMLs had the highest consistency with the 2021 WHO EML for the recommended classes of medicines recommended to treat essential hypertension when compared to other CVD indications. These classes include ACE inhibitors, beta-blockers, thiazide and thiazide-like diuretics and calcium channel blockers. However, the 2021 WHO EML also includes four single pill combinations (SPCs) for the treatment of hypertension. These include combinations of ACE inhibitors and ARBs with thiazides and calcium channel blockers. Since more than two-thirds of hypertension patients may require treatment with more than one antihypertensive medicine, current international hypertension guidelines make strong recommendations to start patients on a combination of pressure lowering medicines preferably in a SPC [26, 27]. Botswana and Lesotho were the only countries to list a SPC in the form of telmisartan/losartan and HCT as recommended in the 2021 WHO EML. Despite the enormity of the hypertension challenge, the South African NEML did not list any SPCs.

Angiotensin receptor blockers (ARBS) were not commonly listed and appeared in less than two-thirds of the SADC formularies even though patients of African origin are well documented to have experienced higher incidence of ACE inhibitor induced cough [28] and updated international guidelines recommend the use of ARBS instead of ACE inhibitors for patients of African origin [26]. Some studies have cited up to a 77% lower incidence of cough and a 66% lower incidence of angioedema in ARBs versus ACE inhibitors [9, 29, 30]. Higher discontinuation rates due to poor tolerance may result in poor CVD outcomes. Furthermore, ARBs were not listed on the South African primary health care NEML and patient’s intolerant to ACE inhibitors may require a change to their regimen or a referral to a hospital. Since most patients travel to the clinic by means of walking [31], referrals to a hospital for medicines that could be provided by a PHC places an additional barrier to accessing healthcare.

Research by Barzagani and colleagues in 2018 reported that ARBS were not listed in any of the low-income countries studied [13], similarly this study showed that recommended SPCs for hypertension were not available in SADC countries. Pricing and availability of these medicines could be a potential barrier to being listed on NEMLs. However, there is evidence to show that the addition of a medicine to existing NEMLs may result in reduction of price and improve cost-effectiveness of that medicine [9]. A study on the pricing of blood pressure lowering medicines across sub-Saharan Africa in 2010 showed that in general the pricing of these medicines were cheaper if they were included on the respective NEMLs [18].

Sodium nitroprusside which is listed as a complementary medicine on the 2021 WHO EML is usually indicated for the treatment of hypertensive emergencies was not listed on the SA NEML and only listed in four out of the fourteen NEMLs studied. This was also seen in a similar study undertaken in eastern Mediterranean countries where only six out of nineteen countries listed the medicine due to its low tolerability.

### Heart failure, antithrombotic and dyslipidaemia medicines

In general SADC countries consistency was high for ACE inhibitors, MRAs, digoxin, aspirin and loop diuretics, moderate for the recommended beta-blockers (bisoprolol, metoprolol and carvedilol), but low for ARBS and clopidogrel. The South African hospital NEML had 100% consistency with the WHO model EML for heart failure and antithrombotic medicines and the PHC NEML listed all foundation medicines for the treatment of heart failure (ACE inhibitors, beta-blockers, MRAs and loop diuretics).

A previous study studying the selection of essential medicines in LMICs across the world found that approximately a quarter of these countries did not have a statin available on their NEML and this was particularly noted for countries in Africa [13]. In this study, statins appeared on 86% of SADC countries NEMLs with both simvastatin and atorvastatin appearing on both the PHC and hospital NEMLs in South Africa.

### Cardiovascular medicines for the treatment of children

Africa has one of the highest prevalence of heart diseases in children driven by congenital heart disease (CHD) and rheumatic heart disease (RHD) [32, 33]. The 2021 WHO EML for children (2021 WHO EMLc) has only included hypertension and heart failure as priority cardiovascular conditions recommending four medicines in tablet, oral liquid or injectable dosage forms. SADC country NEMLs scored high in consistency for formulations like tablets and injections that could be used for adults and children but poorly for child friendly dosage forms like furosemide and digoxin oral liquid (CS = 21% and 43%, respectively). This study found that most SADC countries that listed digoxin and furosemide as oral liquids had their NEMLs in the form of formularies although the cohort was too small to investigate any associations versus formularies in the form of STGs. South Africa has a comprehensive STG for the treatment of children at hospital level and includes treatment for CHD and RHD. This is also the only standalone paediatric STG available in the SADC region. The STG provides comprehensive paediatric dosing but does not list the dosage forms specific to children. The paediatric STG also does not include digoxin in the formulary at hospital level. Since NEMLs are used to guide public sector medicine procurement, STGs should also include age-appropriate dosage forms to improve access to these medicines.

### Limitations of the study

The study used the WHO essential medicines and health products information portal to access NEMLs, but latest NEMLs were not available for all SADC countries. Even though the study made every attempt to obtain updated NEMLs from other published sources there may be some NEMLs that are not in their current versions. We used the 2021 WHO model EML as a comparator list. Many of the SADC NEMLs were last revised prior to 2021, which introduces a bias in terms of consistency with the current WHO EML. However, the objective of the study was to illustrate the differences using the current WHO model list as a comparator. This study was purely a descriptive study and we did not make any additional inferences between countries, comparator lists and country demographics which may impact on the NEMLs. Due to the small cohort this study did not research the association of GDP and health expenditure with length of the NEMLs as seen in previous studies [11, 13].

## Conclusions

This is the first study to focus on cardiovascular medicines in South Africa’s NEML and their comparison globally to the 2021 WHO model EML as well as regionally to the NEMLs of SADC countries. In general, updating of SADC NEMLs need to occur more frequently in sync with the revision cycle for the WHO model list of essential medicines. Improving the consistency of the South African NEML at primary health care level may have a positive impact on cardiovascular outcomes especially when high burden disease like hypertension is concerned. Including age-appropriate dosage forms in the South African STGs may improve access to medicines formulated for children. LMICs adopting the essential medicine list strategy should consider imposing minimum consistency thresholds to the WHO EML to improve accessibility and availability of CVD medicines. Further research is required on the pricing, availability and accessibility of these medicines to determine the impact of the SA NEML on the health system.

## Supplementary Information

**Additional file 1.** Comparison of Southern African Development Community country National Essential Medicines Lists with the 2021 World Health Organisation Model Essential Medicines List.

## Data Availability

The data supporting the conclusions in this article are included within the article and in Additional file 1: Table S2.

## Abbreviations

ACE: Angiotensin converting enzyme
AIDS: Acquired immunodeficiency syndrome
ARB: Angiotensin receptor blocker
CCB: Calcium channel blocker
CHD: Congenital heart disease
CS: Consistency score
CVD: Cardiovascular disease
EML: Essential medicines list
ESC: European Society of Cardiology
ESH: European Society of Hypertension
GDP: Gross domestic product
HAI: Health Action International
HCT: Hydrochlorothiazide
HIV: Human immunodeficiency virus
LMIC: Low- and middle-income countries
MRA: Mineralocorticosteroid antagonist
NCD: Non-communicable disease
NDOH: National Department of Health
NEML: National essential medicines list
PHC: Primary health care
RHD: Rheumatic heart disease
SA: South Africa
SADC: Southern African Development Community
SPC: Single pill combination
SSA: Sub-Saharan Africa
STG: Standard treatment guideline
UK: United Kingdom
WHO: World Health Organization
