# Supplementary material for: A quantitative comparison of essential cardiovascular medicines from countries in the Southern African Development Community to the WHO model essential medicines list
Source: J Pharm Policy Pract. 2022 Dec 8;15:97. doi: 10.1186/s40545-022-00494-0 (PMC9733348; doi:10.1186/s40545-022-00494-0)
Supplement: Supplementary file 1 — Additional file 1. Comparison of Southern African Development Community country National Essential Medicines Lists with the 2021 World Health Organisation Model Essential Medicines List. [file 40545_2022_494_MOESM1_ESM.pdf]

WHO Model List 2021

Table legend: □ = available X = not available Δ = no information

					South Africa (PHC)	South Africa (Hospital)	Angola	Botswana	Comoros	DRC	Lesotho Health Care Level	Lesotho Hospital Level	Malawi	Mauritius	Madagascar	Mozambique	Namibia	Seychelle	Eswatini	Tanzania	Zambia	Zimbabwe	% EMLs with selected meds
Country GDP per capita in US dollars					13600	13600	6800	17000	617	800	3300	3300	1200	11214	1600	1300	11200	29300	4146	3200	4000	2300	
Health Expenditure per Capita (unadjusted US dollars)					1148	1148	239	871	42	32	276	276	93	653	44	79	869	844	271	137	195	115	
EML version (year published)					2020	2019	2021	2012	na	2020	2005	2017	2015	n.d	2008	2017	2016	2010	2012	2017	2017	2015	
Number of medicines on NEML for treatment of cardiovascular diseases					21	38						31											
Medication	Dosage Form	Strength	Comments	Additional Medicines (not on Model List 2021)																			
12.1 Antianginal Medicines																							
Bisoprolol	Tablet	1.25mg; 5mg	Alt: Metoprolol; Carvedilol;	atenolol[a], propanolol[b] labetalol[c]	□[a]	□[a]	□[a], □[b], □[c]	□[a], □[b]	Δ	□[a]	X	□, □[a], □[b] yes	□[a], □[b]	Δ	□[a]	□	□	□[a]	□[a]	□, □[a]	□, □[a], □[b]	□[a]	43%
Glyceryl Trinitrate	Tablet / (sublingual)	500 mcg			X	□	□	□	Δ	□	X	□	□	Δ	X	□	□	X	X	□	□	□	79%
Isosorbide Dinitrate	Tablet / (sublingual)	5mg	Class effect	isosorbide mononitrate[a]	□, □[a]	□, □[a]	□	□	Δ	□	X	□, □[a]	□	Δ	□	□	□	□	X	□, □[a]	□[a]	□	93%
Verapamil	Tablet	40mg; 80mg	Hydrochloride	amlodipine[a], nifedipine[b], diltiazem[c] nicardipine[d]	□[a]	□[a]	□	□, □[b]	Δ	□	X	□ yes	□[a], □[b]	Δ	□[d]	□	□	X	X	□, □[b]	□[b]	□[b]	50%
				nicorandil																			
12.2 Antiarrhythmic medicines																							
Bisoprolol	Tablet	1.25mg; 5mg	Alt: Metoprolol; Carvedilol;	atenolol[a], propanolol[b] labetalol[c] esmolol[d] sotalol[e]	□[a]	□[a]	□, □[a] yes	□[a], □[b]	Δ	□□[a] yes	X	□, □[a], □[b] yes	□[a], □[b]	Δ	□[a]	□	□	□[a]	□[a]	□[a]	□, □[a], □[b]	□[a]	43%
Digoxin	Tablet/ Inj	62.5mcg;250 mcg			X	□	□	X	Δ	□	X	□	X	Δ	□	□	□	□	X	□	□	□	79%
Epinephrine	Injection	100mcg/ml	10ml ampoule; HCL or acid tartrate	isoprenaline[a] noradrenaline[b]	X	□	□	□	Δ	□	X	□, □[a]	□	Δ	□	□	□	□, □[a]	X	□	□	□	93%
Lidocaine	Injection	20mg/ml	5ml ampoule; HCL		X	□	□	□	Δ	□	X	□	□	Δ	□	□	□	□	X	□	□	□	93%
Verapamil	Tablet / Inj	40mg; 80mg	HCL	amlodipine[a], nifedipine[b], diltiazem[c] nicardipine[d]	X	□	□	□	Δ	□	X	□	□	Δ	□[d]	X	□	□	X	□	□[b]	□	71%
Amiodarone	Tab / Injection	50mg/ml	3ml ampoule; HCL (comp)	flecainide[a] quinidine[b]	X	□	□, □[a]	□	Δ	□ yes	X	□	X	Δ	□	□	□	□	X	□	□, □[b]	X	79%
				mexilitine																			
				propafenone																			
				ibutilide																			
				moricizine																			
12.3 Antihypertensive medicines																							
Amlodipine	Tablet	5mg	Maleate,mesylate,besylate;class effect	verapamil[a],nifedipine[b],diltiazem[c] nicardipine[d] nimodipine[e] nitrendapine[f] felodipine	□	□	□, □[a], □[b],	□, □[a], □[b],	Δ	□, □[d]	X	□[a], □[b]	□, □[b], □[e]	Δ	X	□, □[b]	□	□[a], □[c]	X	□	□, □[b]	□	86%
Bisoprolol	Tablet	1.25mg; 5mg	Alt:Atenolol;Metoprolol;Carvedilol	atenolol[a], propanolol[b] labetalol[c] esmolol[d]	□[a]	□, □[a], □[c]	□, □[a], □[b], □[c]	□[a], □[b]	Δ	□	X	□, □[a], □[b], □[c]	□[a], □[b]	Δ	□[a], □[b]	□, □[a]	□	□[a], □[b], □[c]	□[a]	□, □[a], □[b], □[c]	□[a], □[b], □[c]	□[a]	100%
Enalapril	Tablet	2.5mg; 5mg	Hydrogen maleate; class effect	Captopril[a], perindopril[b], lisinopril[c] ramipril[d]	□	□	□, □[a]	□	Δ	□, □[a]	X	□[a], □[c]	□, □[a]	Δ	□[a]	□[c]	□	□[c]	□[a]	□, □[a], □[b]	□[a], □[c]	□	100%
Hydralazine	Tablet	25mg; 50mg	HCL; pregnancy induced hypertension	dihydralazine[a]	X	X	□	□, □[a]	Δ	□[a]	X	□[a]	□	Δ	X	□	□	□	X	□	□	X	71%
Hydrochlorothiazide	Tablet	12.5mg; 25mg	Alt:Chlorothiazide;Chlorthalidone;Indapamide	Bendrofluazide[a],Indapamide[b],+amiloride[c],spironolacton,furosemide[e], torasemide[f] metolazone[g]	□, □[d]	□, □[d]	□, □[d]	□[a], □[b], □[c]	Δ	□, □[b],	□	□, □[b], □[e]	□, □[a]	Δ	□, □[d]	□, □[c]	□	□[a]	□	□[a], □[b], □[e],	□, □[c], □[e]	□	93%
Lisinopril + Amlodipine	Tablet	10mg/5mg; 20mg/5mg; 20mg/10mg	Single pill combination therapy; class effect		X	X	X	X	Δ	X	X	X	X	Δ	X	X	X	X	X	X	X	X	0%
Lisinopril + hydrochlorothiazide (HCT)	Tablet	10mg/2.5mg; 20mg/12.5mg/ 20mg/25mg	Single pill combination therapy; class effect		X	X	X	X	Δ	X	X	X	X	Δ	X	X	X	X	X	X	X	X	0%
Losartan	Tablet	25mg; 50mg; 100mg	Class effect	valsartan[a] telmisartan[b] candisartan[c]	X	□	X	□[b]	Δ	□ yes	X	□ yes	X	Δ	X	X	□	□	X	□	□	X yes	65%
Methyldopa	Tablet	250mg	Pregnancy induced hypertension		X	□	□	□	Δ	□	X	□ yes	□	Δ	□	□	□	□	□	□	X	X yes	93%
Telmisartan + amlodipine	Tablet	40mg/5mg; 80mg/5mg; 80mg/10mg	Single pill combination therapy; class effect	valsartan + amlo[a]	X	X	X	X	Δ	X	X	X	X	Δ	X	X	X	X	X	X	X	X	0%
Telmisartan + HCT	Tablet	40mg/12.5mg; 80mg/12.5mg; 80mg/25mg	Single pill combination therapy; class effect	losartan + HCT [a]	X	X	X	□	Δ	X	X	□[a] yes	X	Δ	X	X	X	X	X	X	X	X	14%
Sodium Nitroprusside	Powder for inf.	50mg / amp	Complementary		X	X	□	□	Δ	□ yes	X	X	X	Δ	X	□	X	X	X	□	X	X	36%
				Prazosin[a], doxazocin[b]	X	X	□[a]	□[b]	Δ	X	X	X	□[a]	Δ	X	X	X	X	X	X	□[a]	□[a], □[b]	36%
				Minoxidil, 5mg, tablet, Urapidil[b] phentolamine[c]	X	X	X	□	Δ	X	X	X	X	Δ	X	X	X	X	X	X	X	X	
				Resepine, 250 micrograms, tablet	X	X	X	X	Δ	X	X	X	□	Δ	X	X	X	X	X	X	X	X	
				Pentoxyphylline					Δ									□					
				Clonidine Tablet 100mcg (C)	X	X	X	X	Δ	X	X	X	X	Δ	X	X	X	□	X	X	X	X	
12.4 Heart Failure Medicines																							
Bisoprolol	Tablet	1.25mg; 5mg	Alt: Metoprolol; Carvedilol;	atenolol[a], propanolol[b] labetalol[c] esmolol[d]	□	□	□	□	Δ	X	X	□, □[a], □[b] yes	X	Δ	□[a], □[b]	□	□	□	X	□	□	□[a], □[b] yes	71%
Digoxin	Tablet	62.5mcg;250 mcg			X	□	□	□	Δ	□	X	□	□	Δ	□	□	□	□[c]	X	□	□	□	93%
Enalapril	Tablet	2.5mg; 5mg	Hydrogen maleate; class effect	Captopril[a], perindopril[b], lisinopril[c] ramipril[d]	□	□	□	□	Δ	□	X	□, □[a], □[b], □[c]	□	Δ	□[a]	X	□	□	□[a]	□, □[a], □[b]	□, □[a]	□	93%
Furosemide	Tablet/injection	40mg	Class effect	Torsemide[a]	□	□	□	□	Δ	□ yes	X	□	□	Δ	□	□	□	□	□	□, □[a]	□	□	100%
Hydrochlorothiazide	Tablet	25mg	Alt:Chlorothiazide;Chlorthalidone;Indapamide	Bendrofluazide[a],Indapamide[b],+amiloride[c], metolazone[d]	□	□	□ yes	X	Δ	□	□	□	□[d]	Δ	X	□	X	X	X	□, □[d]	□, □[d]	□	57%
Losartan	Tablet	25mg; 50mg; 100mg	Class effect	valsartan[a] telmisartan[b] candesartan[c]	X	□	X	X	Δ	X	X	□ yes	X	Δ	X	X	□	□	X	□, □[c]	□	X	43%
Spironolactone	Tablet	25mg		eplerenone[a]	□	□	□	X	Δ	X	X	□ yes	□	Δ	□	□	□	X	□	□, □[a]	□	□	79%
Dopamine	Injection	40mg/ml	5ml vial; HCL; complementary listing	dobutamine[a],	X	□[a]	□	□	Δ	□	X	X	□	Δ	□	X	□	□, □[a]	□, □[a]	□, □[a]	□	X yes	86%
12.5 Antithrombotic medicines																							
12.5.1 Anti-platelet medicines																							
Acetylsalicylic acid	Tablet	100mg			□	□	□	□	Δ	□	X	□	□	Δ	□	X	□	□	X	□	□	□	86%
Clopidogrel	Tablet	75mg; 300mg			X	□		□	Δ	□ yes	X	X	X	Δ	X	X	□	□	X	□	X	X yes	43%
12.5.2 Thrombolytic medicines																							
Alteplase	Powder for inj.	10mg; 20mg; 50mg	Vial; complementary listing	Tenecteplase[a] reteplase[b]	X	□	X	X	Δ	□	X	X	X	Δ	X	X	X	□[b]	X	□, □[a]	X	X	21%
Streptokinase	Powder for inj.	1.5 million IU	Vial; complementary listing		□	□	□	□	Δ	□ yes	X	□	X	Δ	X	X	X	X	X	□	X	□	50%
12.6 Lipid-Lowering Agents																							
Simvastatin	Tablets	5mg; 10mg; 20mg; 40mg	Use in high risk patients; class effect	Atorvastatin[a] rosuvastatin[b]	□, □[a]	□, □[a]	□, □[a]	□, □[a]	□, □[a]	□, □[a]	□, □[a]	□, □[a] yes	□	Δ	X	□	□	□	□	□, □[a], □[b]	X	X	86%
				Fenofibrate[a], gemifibrozil[b], bezafibrate[c]	X	X	X	□[c]	Δ	X	X	X	X	Δ	X	X		X	X	□[a], □[b]	X	X	
Children																							
12.1 Antianginal Medicines																							
12.2 Antiarrhythmic medicines																							
12.3 Antihypertensive medicines																							
Enalapril	Tablet	2.5mg; 5mg	Hydrogen maleate, class effect	Captopril[a], perindopril[b], lisinopril[c]	□	□, □[a]	□	□	Δ	□	X	□, □[a], □[b], □[c]	□	Δ	□[a]	□[c]	□	□	□[a]	□	□[a], □[c]	□	100%
		50mg/5ml		Hydrochlorothiazide oral liquid / 0,5 mg tab	X	□	X	X	Δ	□	X	X	X	Δ	X	X	X	X	X	X	X	X	
				atenolol[a], propanolol[b] labetalol[c]	X	□[a], □[b], □[c]	X	□	Δ	X	X	X	X	Δ	X	Δ	X	□	X	X	□, □[a], □[b]	X	
				Amlodipine 0.2mg, tablet	X	□	X	X	Δ	X	X	X	X	Δ	X	Δ	X	X	X	X	X	X	
				Prazosin, 0.005mg, tablet	X	□	X	X	Δ	X	X	X	X	Δ	X	Δ	X	X	X	X	X	X	
12.4 Heart Failure Medicines																							
Digoxin	Injection	250mcg/ml	2ml ampoule		X	X	□ yes	X	Δ	□	X	□ yes	□	Δ	Δ	□	□	□	X	□	□	□	71%
Digoxin	Oral Liquid	50mcg/ml			X	X	□	□	Δ	□	X	X	X	Δ	Δ	□	□	□	X	X	X	X	43%
	Tablet	62.5mcg;250 mcg			X	X	□ yes	□	Δ	□	X	□	X	Δ	□	□	□	□	X	□	□	□	79%
Furosemide	Injection	10mg/ml	2ml ampoule; class effect		□	□	□	□	Δ	X	X	□	□	Δ	Δ	□	□	□	X	□	□	□	79%
	Oral Liquid	20mg/5ml			X	X	X	□	Δ	X	□	x	X	Δ	Δ	□	□	X	X	X	X	□ no	21%
	Tablet	40mg			X	□	□ yes	□	Δ	X	X	□	X	Δ	□	□	□	□	□	□	□	□	86%
Dopamine	Injection	40mg/ml	5ml vial; HCL; complementary listing	dobutamine[a],	X	□[a]	□	□	Δ	□	X	X	□	Δ	□	X	□	□	X	□, □[a]	□	X yes	79%
				Bisoprolol, 1mg/ml, oral liquid	X	X	□	X	Δ	X	X	X	X	Δ	X	X	X	X	Δ	X	X	X	
				Spironolactone, 1mg, tablet	X	□	X	X	Δ	X	X	□	□	Δ	X	Δ	X	X	Δ	X	X	X	
12.5 Antithrombotic medicines																							
12.5.1 Anti-platelet medicines																							
12.5.2 Thrombolytic medicines																							
12.6 Lipid-Lowering Agents																							
Comparison Score (CS) - combined adult and paed					17/43 = 35%	70%	79%	74%		77%	7%	77%	51%	0%	44%	67%	81%	65%	26%	84%	67%	65%	Average = 66%
Comparison Score (CS) - adult only					15/35 = 43%	71%	77%	71%		77%	6%	80%	51%	0%	43%	63%	77%	60%	26%	86%	66%	63%	Average = 65%
Comparison Score (CS) - paeds only					2/8 = 25%	50%	88%	89%		75%	13%	63%	50%	0%	50%	88%	100%	88%	25%	75%	75%	75%	Average = 71%
